# Biosynaptic devices based on chicken egg albumen:graphene quantum dot nanocomposites

**DOI:** 10.1038/s41598-020-57966-z

**Published:** 2020-01-27

**Authors:** Sihyun Sung, Jae Hyeon Park, Chaoxing Wu, Tae Whan Kim

**Affiliations:** 1 Department of Electronics and Computer Engineering, Hanyang University, Seoul, 04763 South Korea; 2 College of Physics and Information Engineering, Fuzhou University, Fuzhou, 350108 China

**Keywords:** Engineering, Materials science

## Abstract

Biosynaptic devices based on chicken egg albumen (CEA):graphene quantum dot (GQD) hybrid nanocomposites were fabricated to achieve stable synaptic behaviors. Current-voltage (I-V) curves for the biosynaptic devices under consecutive negative and positive voltage sweeps showed clockwise pinched hysteresis, which is a critical feature of a biological synapse. The effect of the GQD concentration in the CEA layer on the device performance was studied. The retention time of the biosynaptic devices was relatively constant, maintaining a value above 10^4^ s under ambient conditions. The carrier transport mechanisms of the biosynaptic devices were described and analyzed on the basis of the slopes of the I-V curves and their fittings.

## Introduction

Due to the limitation on the speed of data transfer between the memory and the central processing unit, neuromorphic computing is faced with a bottleneck when the von Neumann computing architecture is used^1,2^. The use of nanoscale integrated circuitry based on a biomimetic brain to operate a neuromorphic system is an emerging research field because such platforms offer high-speed processing and improved energy efficiency. These demands have prompted the development of two-terminal memristors as promising candidates for artificial synapses because they can emulate synaptic plasticity with low power consumption. Many two-terminal synaptic devices are required, for reduced production cost, to mimic the complex human brain^2–6^.

Nowadays, various oxide-based materials have been reported as components for resistive switching devices. The resistive switching mechanism of oxide-based devices is observed in the formation and the rupture of conducting filaments in the dielectric due to the application of an electrical pulse^7–10^. In addition, the memristors fabricated with organic materials are based on the resistive switching mechanism. The synaptic characteristics of memristors fabricated using organic/inorganic hybrid nanocomposites have been intensively investigated because such devices offer the advantages of simple fabrication, low cost, high flexibility, and low power consumption. When synaptic devices are fabricated utilizing inorganic/organic nanocomposites, matrix materials with charge-storage capability are typically deposited by using a spin-coating method^11–14^.

Recently, a variety of emerging materials have been introduced to implement artificial neurons and artificial synapses^15,16^. Among the various biomaterials, chicken egg albumen (CEA) has been extensively used for potential applications in memristive devices, transistors, and synaptic devices^17–21^. CEA has recently been considered as a novel candidate owing to its promising applications in devices with human-friendly properties and in next-generation devices. The proteins in albumen will denature when a large amount of heat energy is applied in the devices. The denaturation of the proteins in CEA reduces the probability of oxygen scattering and changes the paths of oxygen diffusion, resulting in an increase in the possibility of forming and rupturing conductive filaments.

Graphene quantum dots (GQDs), which are included in the ultrafine graphene family, show excellent properties of superior mechanical flexibility, high work function, and excellent charge-storage capabilities, making them excellent candidates for potential electroluminescent applications in electronics and optoelectronics^22–25^. In addition, GQDs cause strong quantum-confinement and significant edge effects in nanoscale devices^26^. The crystallographic orientation of the graphene edges markedly affects the electronic properties of the GQDs, including the formations of a mobility gap and a Coulomb barrier^27^. Therefore, hybrid nanocomposites based on GQDs, which have a remarkable charge-storage capability, embedded in a polymer layer, which has a low-dielectric constant, are very effective active layers in memristive and synaptic devices^14,24^.

This paper presents data for the electrical properties and the operating mechanisms of biosynaptic devices using CEA/GQD nanocomposites as an active layer. The current-voltage (I-V) characteristics of the synaptic devices indicated the presence of pinched hysteresis under consecutive negative and positive voltage sweeps, and the electrical characteristics were found to depend significantly on the GQD concentration. Furthermore, the carrier transport and the operating mechanisms of the biosynaptic devices were identified and then described on the basis of the slopes of the linear portions of the curves fitting the I-V data.

## Methods

**Figure 1:**
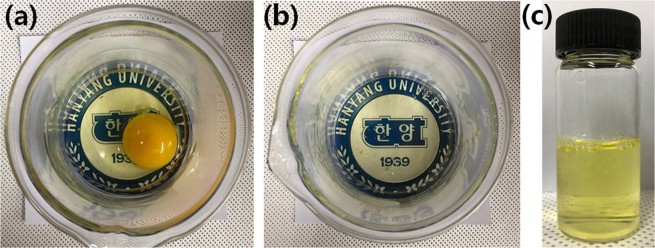
Optical images of (**a**) the entire egg consisting of an egg white and an egg yolk, (**b**) the egg albumen liquid, and (**c**) the egg albumen:GQD solution.

Figure 1 shows the solution used for the biosynaptic devices investigated in this work. Unfertilized chicken eggs were used to prepare the devices in this study. The entire egg consisting of egg white and egg yolk was separated by using a steel-mesh spoon, as shown Fig. 1(a,b). The separated CEA liquid was mixed with a GQD solution (ACS MATERIAL) in volume ratios of 0, 5, 10, 15 and 20%, as shown Fig. 1(c), followed by an ultrasonic process for 15 min at room temperature^18^.

Indium-tin-oxide (ITO)-coated glass substrates were cleaned ultrasonically in acetone, methanol, and de-ionized (DI) water for 30 min each. After the chemically cleaned ITO glass substrates had been dried by using N_2_ gas with a purity of 99.99%, the biosynaptic devices with GQDs embedded in the CEA layer were fabricated on ITO glass substrates. The CEA:GQD thin layers were formed on the ITO substrates by using a spin-coating method at spin-coating speeds of 500 rpm for 3 s, 1500 rpm for 5 s, 4000 rpm for 30 s, 1500 rpm for 5 s, and 500 rpm for 3 s in series at room temperature. Then, because the protein denatures during high-temperature treatment, the devices were annealed at 120°C for 20 min, resulting in improved electrical performance^18^. The top Al electrodes, each with a thickness of 200 nm and a diameter of 1 mm, were deposited through a metal mask onto the CEA:GQD layer by using thermal evaporation at a system pressure of 1 × 10^−6^ Torr.

The structural properties of the Al/CEA:GQD/ITO devices were characterized by using scanning electron microscopy (SEM, Verios G4 UC). All electrical measurements on the devices were performed by using a semiconductor characterization system (Keithley 2400) at 300 K.

## Results and Discussion

**Figure 2:**
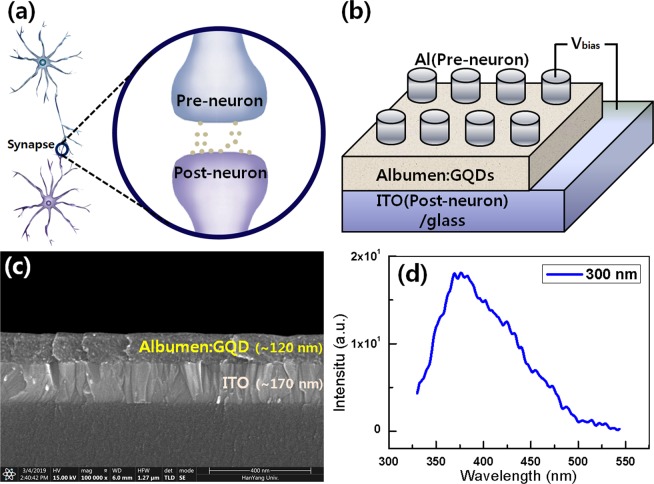
Schematic diagrams of (**a)** the neuron and the synapse and (**b**) the ITO/CEA:GQD/Al device. (**c**) Cross-sectional SEM image of the ITO/CEA:GQD/Al device. (**d**) Photoluminescence spectrum of the GQDs under 300-nm excitation.

Figure 2(a,b) show schematics of the structures of the biological synapse and the synaptic device used in this work, respectively. In the synaptic devices with a ITO/CEA:GQD/Al structure, the Al (top) and the ITO (bottom) electrodes work as the pre-synaptic and the post-synaptic neurons, respectively. Figure 2(c) shows a cross-sectional SEM image of the CEA:GQD nanocomposite formed on an ITO-coated glass substrate. The thickness of the CEA:GQD nanocomposite film was approximately 120 nm. Figure 2(d) presents the photoluminescence (PL) spectrum for the GQDs, which clearly indicates the existence of GQDs in the active layer. When the GQDs are excited at 300 nm, they show a blue emission peak at 370 nm. The PL mechanism of GQDs can be explained by the size of the GQDs, surface chemical groups, and doping atoms^28^.

**Figure 3:**
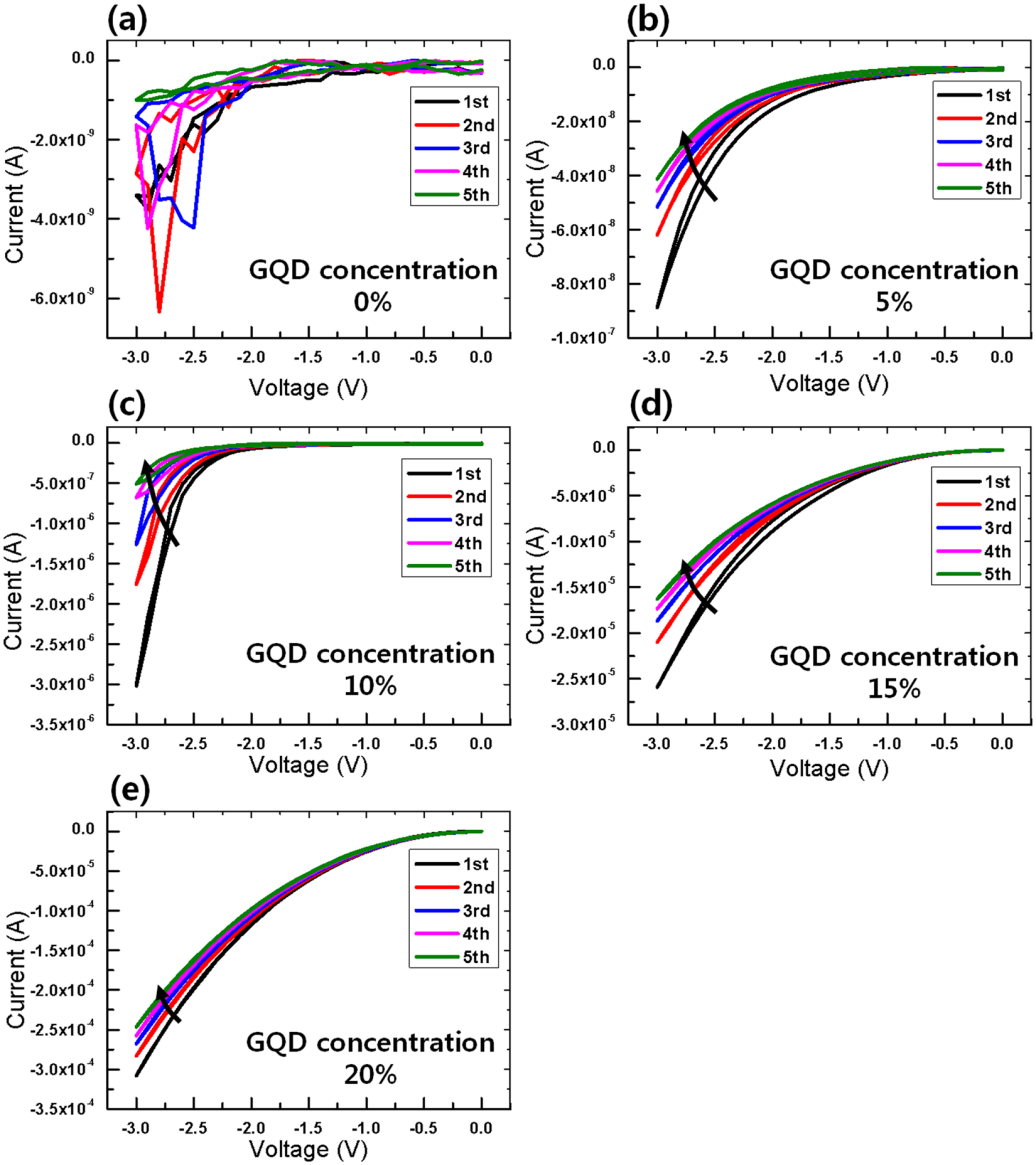
I-V curves of the ITO/CEA:GQD/Al devices during five voltage sweeps for GQD concentrations of (**a**) 0, (**b**) 5, (**c**) 10, (**d**) 15, and (**e**) 20%.

Figure 3 shows the I-V curves for the biosynaptic devices with the ITO/CEA:GQD/Al structure for GQD concentrations of (a) 0, (b) 5, (c) 10, (d) 15, and (e) 20%. The ITO electrode was grounded, and the voltage was swept 5 times from 0 to −3 to 0 V. The concentration of the GQDs embedded in CEA can significantly affect the electrical characteristics. The biosynaptic devices were focused on the I-V curves under applied negative voltages because the resistive switching devices based on CEA are operated as a Set process under applied negative voltages and a Reset process under applied positive voltages^18^. Overall, the resistance of the device decreased with increasing concentration of GQDs. Because a GQD is a highly conductive material, the conductivity of the CEA:GQDs nanocomposite layer increased. The synaptic devices with only CEA (no GQDs) in the active layer exhibited low conductance, as shown Fig. 3(a). The device exhibited irregular and unstable electrical properties during the five sweeps. However, the other devices, those with GQDs, exhibited a clockwise pinched hysteresis behavior. Figure 3(b) shows the I-V curves of the synaptic device with a 5% GQD concentration. The conductance consistently decreased with increasing number of voltage sweeps. The presence or absence of GQDs has a great influence on the characteristics of the synapse because the device with a 5% GQD concentration has more stable synaptic behaviors than the device with a 0% GQD concentration. For the device with a 10% GQD concentration, the conductance decreased with increasing number of dual voltage sweeps, as shown in Fig. 3(c). The drastic changes in the currents in the I-V curves at −3 V for the first, second, third, fourth and fifth I-V curves for that device were −3.0 × 10^−6^, −1.75 × 10^−6^, −1.26 × 10^−6^, −6.74 × 10^−7^, and −5.06 × 10^−7^ A, respectively. The rate of variation in the conductance with increasing number of applied voltage sweeps is a natural feature of electronic synapses. Figure 3(d,e) show I-V curves for the CEA devices with 15 and 20% GQD concentrations, respectively. Although both devices also exhibit clockwise pinched hysteresis behavior, the rates of variation in the conductance were smaller than that for the device with a 10% GQD concentration.

**Figure 4:**
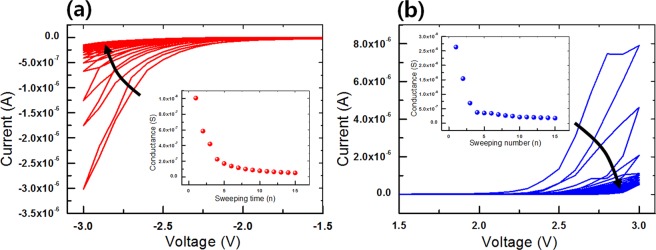
I-V curves of the ITO/CEA:GQD/Al devices under (**a**) dual negative voltage sweeps and under (**b**) dual positive voltage sweeps, with the insets showing the conductance as a function of the number of sweeps for those devices.

Figure 4 shows more detailed electrical characteristics for the optimized ITO/CEA:GQD/Al device, i.e., the device with a 10% GQD concentration. The rate of variation in the conductance after five consecutive voltage sweeps is the largest value compared with the other devices with GQD concentrations of 0, 5, 15, and 20%. The I-V curves when 15 consecutive negative voltage sweeps (0 to −3 to 0 V) were applied to the synaptic device are shown in Fig. 4(a). The inset shows the conductance at −3 V as a function of the number of applied negative voltage sweeps. Under dual negative voltage sweeps, the synaptic device showed a clockwise pinched hysteresis behavior. The electrical characteristics of the device showed a high-resistance state (HRS) in the forward sweep and a relatively low-resistance state (LRS) in the reverse sweep. While consecutive negative voltage sweeps were being applied, the current at −3 V increased from −3.0 × 10^−6^ A (1st sweep) to −1.48 × 10^−7^ A (15th sweep). This means the conductance decreases with increasing number of dual negative voltage sweeps. This behavior is similar to the depression behavior in a biologic synapse. Figure 4(b) shows the I-V curves of the synaptic devices under consecutive positive voltage sweeps (0 to 3 to 0 V). The inset shows the conductance at 3 V as a function of the number of applied positive voltage sweeps. A clockwise pinched hysteresis behavior of the synaptic device was also seen under dual positive voltage sweeps. The current in the synaptic device continually decreased with increasing number of voltage sweeps. The current in the synaptic device under consecutive positive voltage sweeps at 3 V decreased from 7.9 × 10^−6^ A (1st sweep) to 5.2 × 10^−7^ A (15th sweep). As with the negative voltage bias, the conductance decreased with increasing number of voltage sweeps. When the I-V curves for the negative and the positive voltage stimulations are compared, the variations in the currents are found to be similar, indicating that the synaptic device when undergoing positive voltage sweeps also shows a depression behavior.

**Figure 5:**
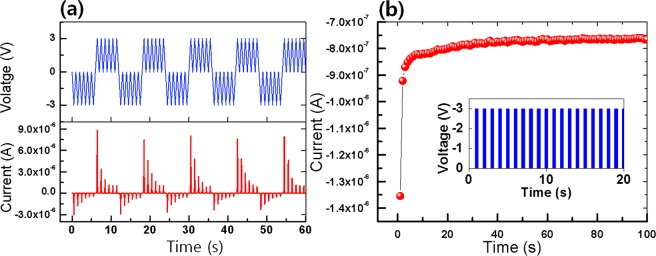
(**a**) Current and voltage as functions of time to establish the endurance characteristics when consecutive voltage pulses are applied to the device. (**b**) I-t curves for the device under a pulsed voltage. The inset in (**b**) presents the pulsed voltage applied to the device.

Figure 5(a) shows a series of voltage stimulations with six pairs of negative voltage followed by positive voltage being applied to the ITO/CEA:GQD/Al synaptic device. The Set and the Reset conditions can be seen in the V-t and the I-t data. Thus, we can conclude that the synaptic device is rewritable with repeated switches between low-resistance and high-resistance states. Figure 5(b) presents the I-t curves when 100 cycles of the pulsed voltage (3 V, 100 ms) are applied to the ITO/CEA:GQD/Al synaptic device. The inset of Fig. 5(b) presents the pulsed voltage applied to the biosynaptic device. The current under the pulsed voltage increases from −1.35 × 10^−6^ to −7.66 × 10^− 7^ A. This behavior is the same as the long-term depression (LTD) of synaptic characteristics.

**Figure 6:**
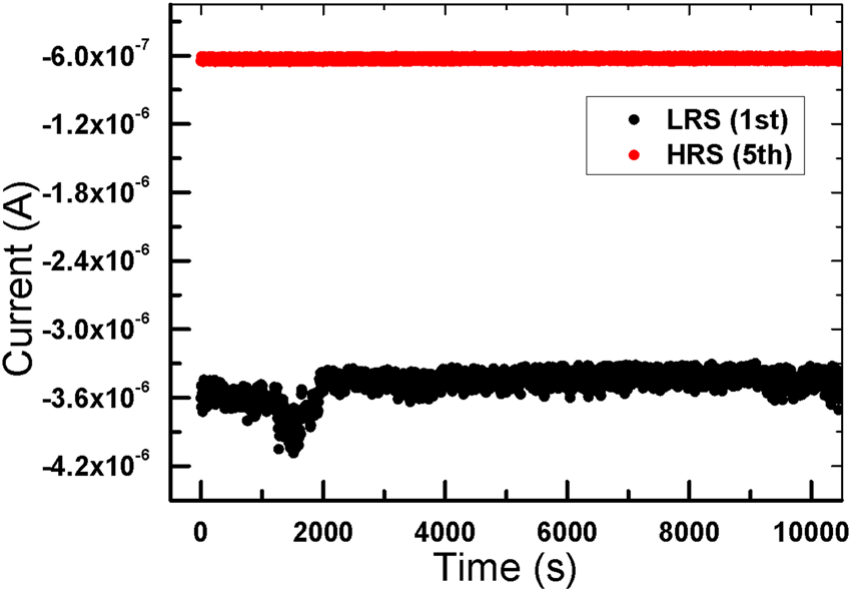
Retention characteristics (I-t) of the device after the first and the fifth voltage sweeps at a reading voltage of −3 V.

The retention abilities of the ITO/CEA:GQD/Al synaptic devices were determined by measuring the performances of the low-resistance and the high-resistance states of the devices under ambient conditions, and the results are shown in Fig. 6. The measurements were made at room temperature, and an initial reading voltage of −3 V (1st) and −3 V after four consecutive negative voltage sweeps (5th) had been applied to the device. When the I-t curves for the devices were extrapolated to 10^4^ sec, they were found to maintain a constant current, which is indicative of the long-time stability of the devices.

**Figure 7:**
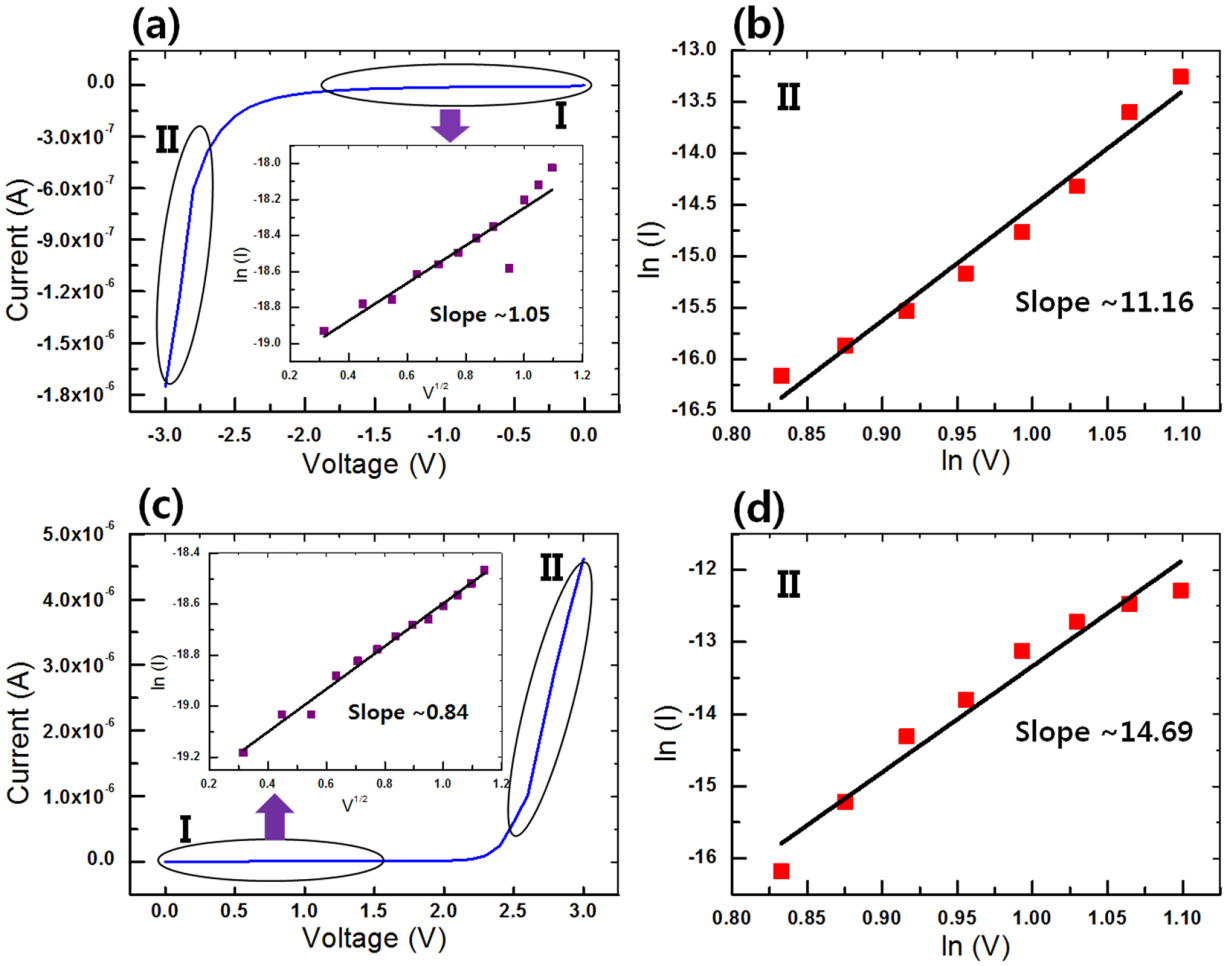
(**a**) I-V curve of the ITO/CEA:GQD/Al device when a negative voltage from 0 to −3 V is applied in the 2nd sweep. The inset shows a fitting of the I-V data with a ln (I) versus V^0.5^ curve for the device under a negative voltage from 0 to −1.2 V (region I in (a)). (**b**) Fitting of the I-V data with a ln (I) versus ln (V) curve under a negative voltage from −2.3 to −3 V (region II in (**a**)). (**c**) I-V curve of the ITO/CEA:GQD/Al device when a positive voltage from 0 to 3 V is applied in the 2nd sweep. The inset shows a fitting of the I-V data with a ln (I) versus V^0.5^ curve for the device under a positive voltage from 0 to 1.3 V (region I in (**c**)). (**d**) Fitting of the I-V data with a ln (I) versus ln (V) curve under a positive voltage from 2.3 to 3 V (region II in (**c**)).

I-V fittings were performed in order to clarify the carrier transport mechanisms in the ITO/CEA:GQD/Al synaptic devices, and the results are shown in Fig. 7. The thermionic emission (TE) and the space-charge-limited current (SCLC) models were used according to the following equations^29,30^:

1 $${\rm{I}}\propto {\rm{A}}{T}^{2}\exp \,\left[-\frac{q\phi }{kT}+q{\left(\frac{{q}^{3}V}{4\pi \varepsilon }\right)}^{1/2}\right],$$

2 $${\rm{I}}\propto {V}^{\alpha },$$

respectively, where I, V, A, T, ε, φ, k, and q represent the current, applied voltage, Richardson’s constant, absolute temperature, dielectric permittivity, barrier height, Boltzmann’s constant, and electronic charge, respectively.

Figure 7(a) shows the I-V curve when a negative voltage (0 to −3 V) in the 2nd sweep was applied to the electronic synaptic device. The inset presents the fitted graphs under a negative voltage sweep. The ln (I) versus V^1/2^ curve is linear at voltages below −1.2 V (region I), indicating that TE dominates the carrier transport in that region. The slope of the ln (I) versus ln (V) curve for positive voltage from −2.3 to −3 V (region II) is about 11.16, as shown in Fig. 7(b). The carrier transport in the synaptic device is dominated by SCLC conduction, which results from charge capture by the GQDs. The gradual variation in the conductance is attributed to the introduction of GQDs. As is well known, GQDs show superior charge-storage capabilities for potential applications in electronics. The charge-storage ability is derived from charge trapping by the GQDs, which affects the material transport^31–34^. The electric carriers under the first negative pulse are injected from the Al electrode into the albumen:GQD structure via TE. The current is the highest in that case. The transport mechanism has been modulated by values of the voltage sweeping times. This shows multiple states resulting from gradual oxidation of CEA or generation of more Fe ions^18,35^. Also, some of the injected carriers are captured by the GQDs, resulting in the formation of space charges. These space charges can induce an internal reverse electric field, which accordingly weakens the external electric field and leads to the inhibition of charge injection. Thus, the conductivity of the e-synapse decreases, and the e-synapse tends to switch from the LRS to the HRS. Figure 7(c,d) show the fitted graphs under positive consecutive voltage (0 to 3 V) for the 2nd sweep. A linear relationship is seen between ln (I) and V^1/2^ at voltages below 1.3 V (region I), as shown in the inset of Fig. 7(c), and a linear relationship is seen between ln (I) and ln (V) at voltages from 2.3 to 3 V (region II), as shown in Fig. 7(d). The positive voltage can release the previously trapped charges, leading to the recovery of the conductivity. However, the injected carriers under the ensuing positive voltage are captured by the GQDs again, resulting in a switching operation from the LRS to the HRS. Worth noting is that when the polarity of the voltage reverses again, the above process is repeated. As a result, the e-synapse is expected to exhibit high operation stability.

## Conclusion

The I-V curves for ITO/CEA:GQD/Al devices with various concentrations of GQDs at room temperature showed that the variation rates in the currents in the devices could be maximized at a GQD concentration of 10%, resulting in improved device performance. The I-V characteristics of the biosynaptic devices exhibited a clockwise pinched hysteresis behavior similar to that shown by an artificial e-synapse. The biosynaptic devices demonstrated stable synaptic performance with retention times above 10^4^ s without significant electrical degradation. The carrier transport mechanisms in the biosynaptic devices were dominated by TE and SCLC conduction.
